# Supplementary material for: Structures of liganded glycosylphosphatidylinositol transamidase illuminate GPI-AP biogenesis
Source: Nat Commun. 2023 Sep 8;14:5520. doi: 10.1038/s41467-023-41281-y (PMC10491789; doi:10.1038/s41467-023-41281-y)
Supplement: Supplementary file 1 — Supplementary Information [file 41467_2023_41281_MOESM1_ESM.pdf]

Title: Structures of Liganded Glycosylphosphatidylinositol Transamidase Illuminate GPI-AP Biogenesis

Author list: Yidan Xu[1,†], Tingting Li[1,†], Zixuan Zhou[2,†], Jingjing Hong[1], Yulin Chao[2], Zhini Zhu[2], Ying Zhang[2], Qianhui Qu[2,*], Dianfan Li[1,*]

Supplementary Information

Supplementary Figs. 1-15

Supplementary Table 1

Movie Still for Supplementary Movies 1-2

Supplementary References 1-5

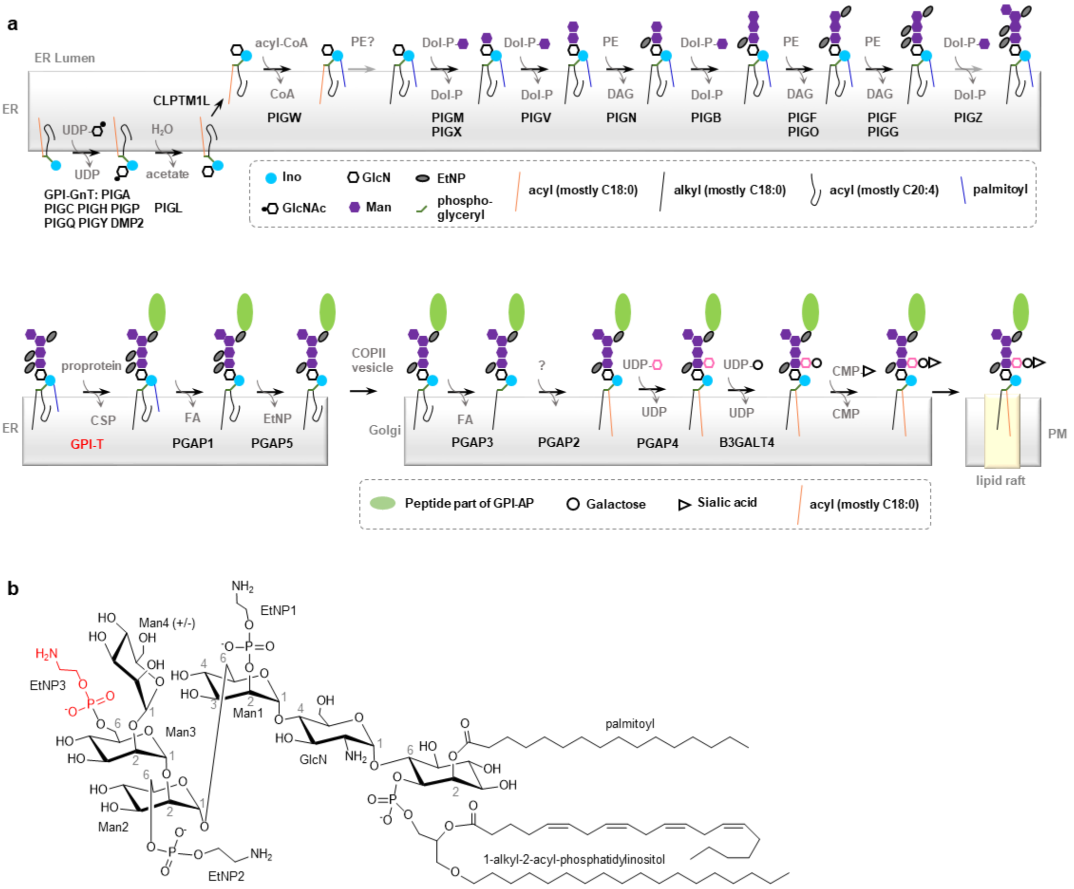

Supplementary Figure 1 | Schematic of GPI-AP biosynthesis. a The multi-step biosynthesis of GPI-AP. GPI is first synthesized by enzymes that catalyze the flipping of lipids[1], and the building and modification of the glycan core. After being attached to proproteins by GPI-T, the GPI moiety is remodeled in both the acyl chain and the glycan residues. Various components of GPI are indicated in dashed boxes. Enzymes and the substrates/products are indicated for each step. The pathway is redrawn based on ref.[2] . **b** Chemical structure of a typical GPI. Various components are labeled. EtNP3, the bridging EtNP for most GPI-APs, is colored red. Relevant carbon atoms are indicated by a number. The phosphatidyl group is drawn as the dominant 1-alkyl, 2-acyl composition[3]. A “+/-” sign indicates the optional Man4 modification for human GPIs. ER, endoplasmic reticulum; EtNP, ethanolamine phosphate; GlcN, glucosamine; Man, mannose; Ino, inositol; PM, plasma membrane.

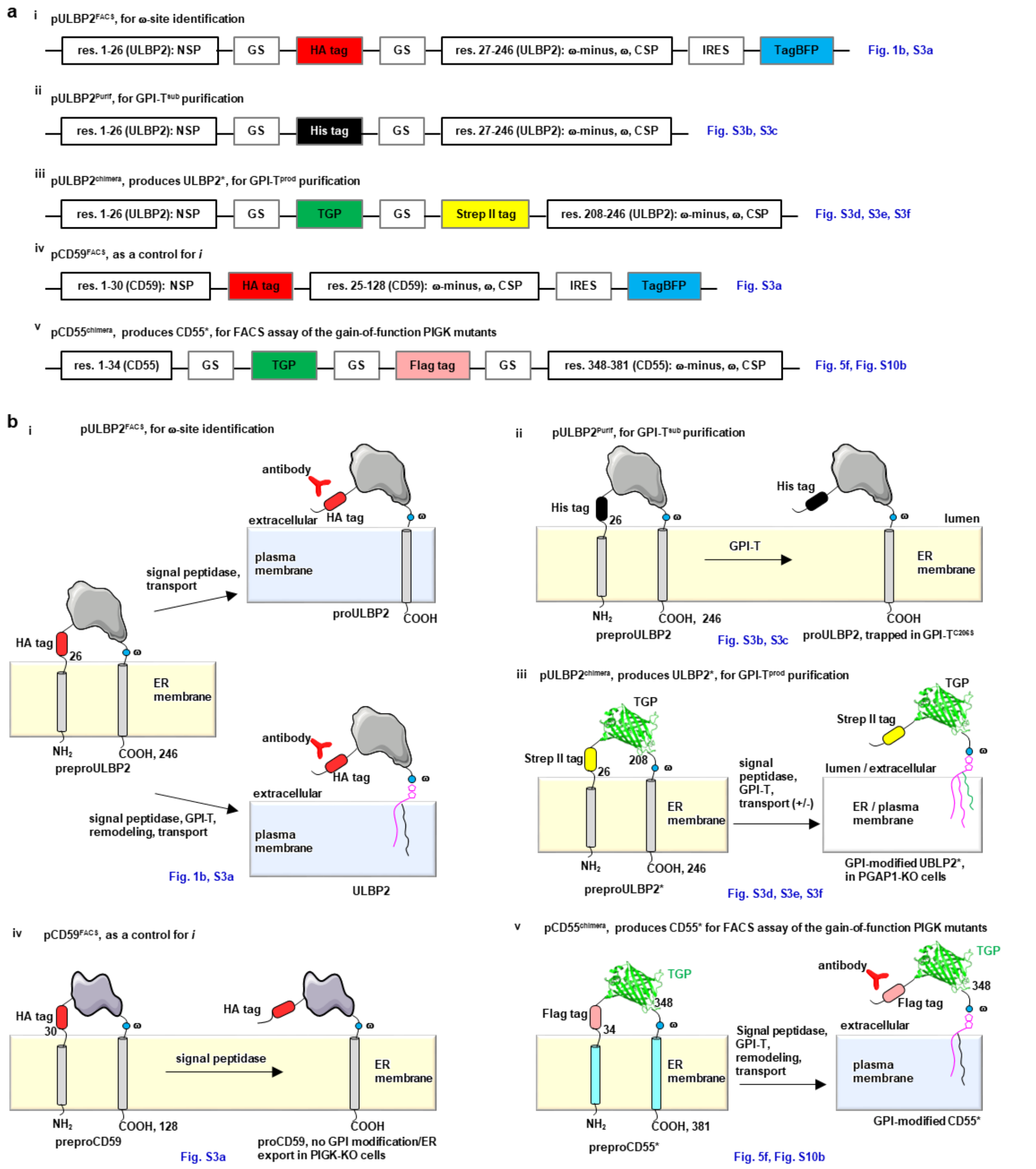

Supplementary Figure 2 | Constructs and processing of recombinant GPI-APs in this study. a Schematic of elements in the recombinant GPI-APs. The name and their purpose are indicated above each construct, while figures citing the constructs are indicated in blue texts. An internal ribosome entry site (IRES) in (**i**) and (**iv**) is used to co-express the GPI-AP and tagBFP, which serves as a gating marker during fluorescence-activated cell sorting (FACS) for the successful expression of GPI-APs. BFP, blue fluorescence protein; GS, glycine-serine linker; HA, hemagglutinin tag; TGP, thermostable green fluorescence protein. NSP/CSP, N-/C-terminal signal peptide. **b** Schematic of the GPI-APs in **a** and their speculated cellular fate. Relevant residues numbering are shown. The glycan part of GPI is indicated by magenta pentagons. The acyl chains of GPI are colored magenta, green (inositol acyl), or black (remodeled *sn*-2). Figures citing the GPI-APs are indicated by blue text. A “+/-”sign in (**iii**) indicates compromised vesicle transport of the triacylated GPI-AP due to the absence of PGAP1. ER, endoplasmic reticulum.

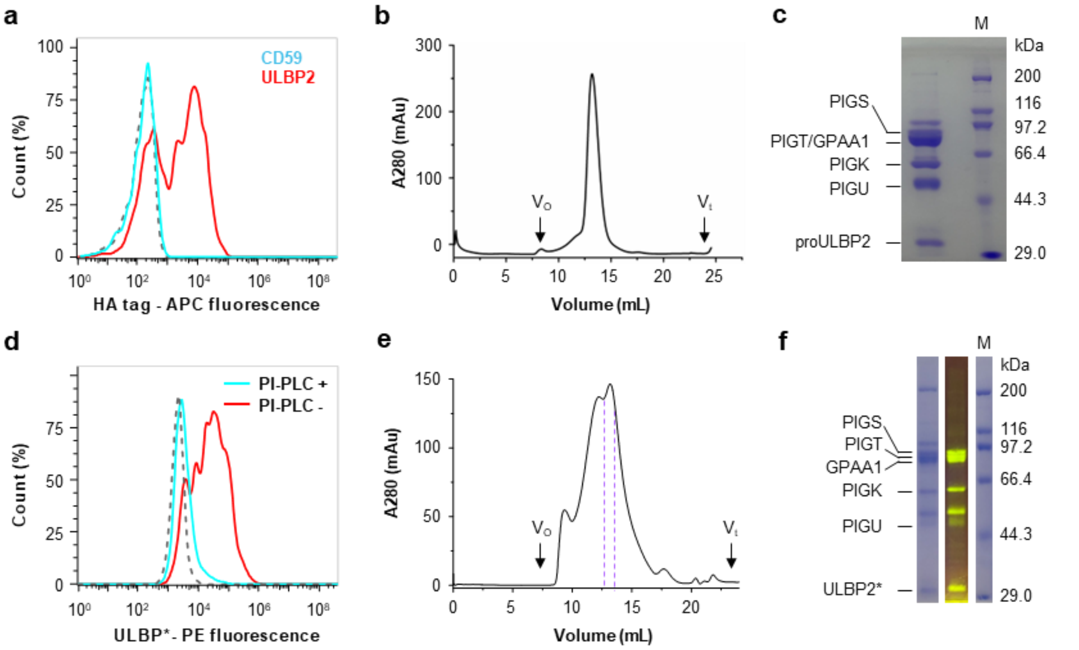

Supplementary Figure 3 | Characterization and purification of the substrates and products complexes. a Evidence for transmembrane helix-mediated surface anchoring of ULBP2. ULBP2 and CD59 were separately recombinantly expressed in PIGK-KO cells with an HA-tag for surface staining. Background staining of the antibody is indicated by a grey dash line. See Supplementary Fig. 15a for gating strategy. **b** Gel filtration profile of GPI-T^{C206S} with proULBP2 bound. V_o, void volume; V_t, total volume. **c** SDS-PAGE of the peak fraction in **b**. PIGS, PIGT, and GPAA1 co-immigrated on the gel. **d** Evidence for GPI-anchoring of the chimeric proULBP2*. The construct contains a thermostable GFP (TGP) with the N- and C-terminal signal peptides from ULBP2 and an HA-tag for staining. Surface staining was performed for HEK293 cells treated with (cyan) or without PI-PLC (red). Grey trace indicates the staining of cells expressing an unrelated TGP-tagged membrane protein. See Supplementary Fig. 15b for gating strategy. **e** Gel filtration profile of the ULBP2* in complex with GPI-T. Dashed lines indicate fractions used for structure determination. **f** Coomassie staining (blue) and in-gel fluorescence (green) of the TGP-containing subunits and ULBP2*. Source data are provided as a Source Data file.

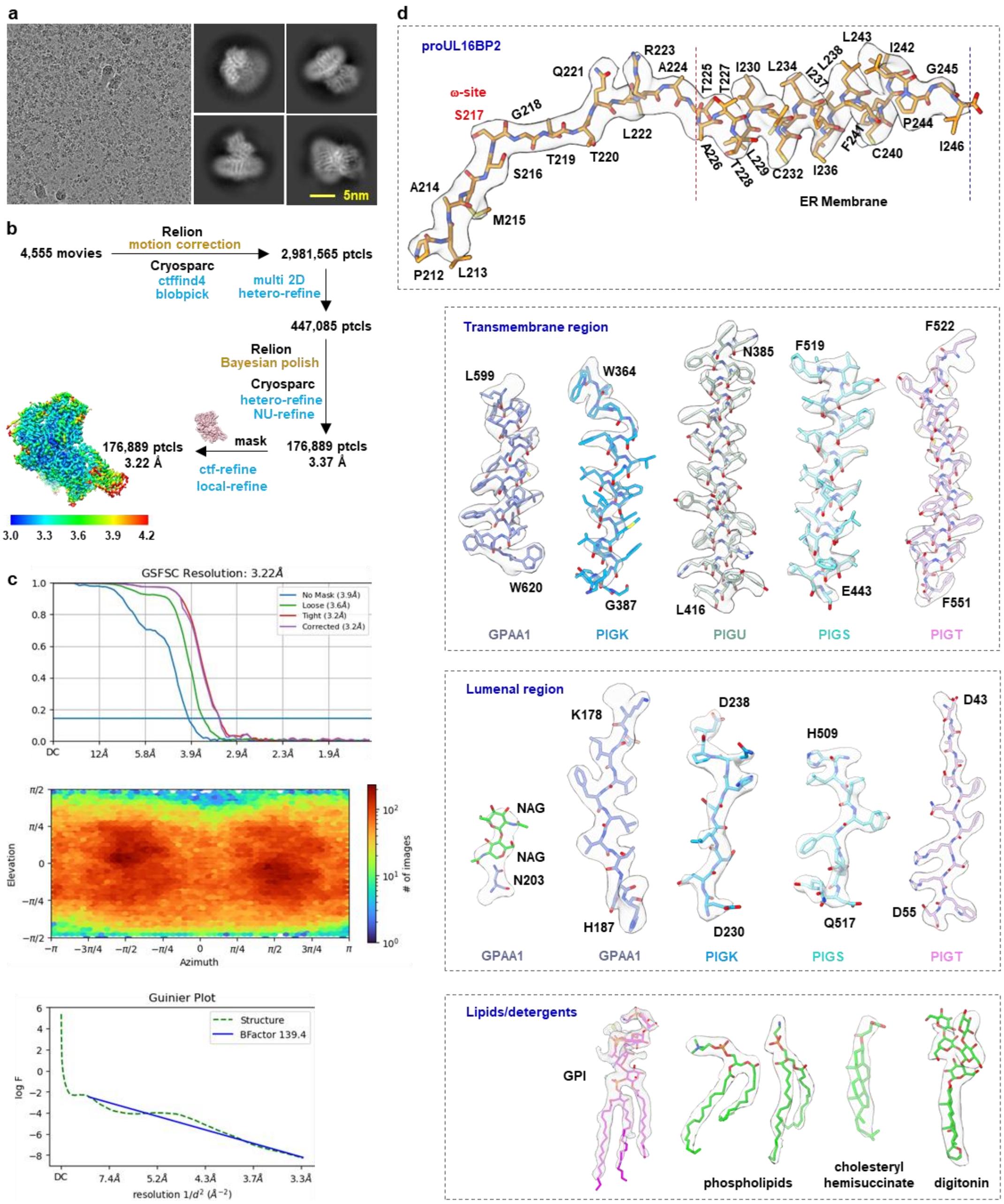

Supplementary Figure 4 | Cryo-EM data processing and density/model fitting exemplary views for GPI-T^{sub}. a Representative cryo-EM micrograph of GPI-T^{sub} and selected 2D class averages. **b** Workflow of the classification and refinement. **c** The nominal resolution of GPI-T was determined by the 'gold-standard' FSC curve using the FSC=0.143 criterion, and angular distribution heatmap at the bottom panel calculated in Cryosparc. Local resolution evaluation was shown on right. **d** Cryo-EM map density and model of representative protein parts, the protein parts that are discussed in the main text, lipids and detergents.

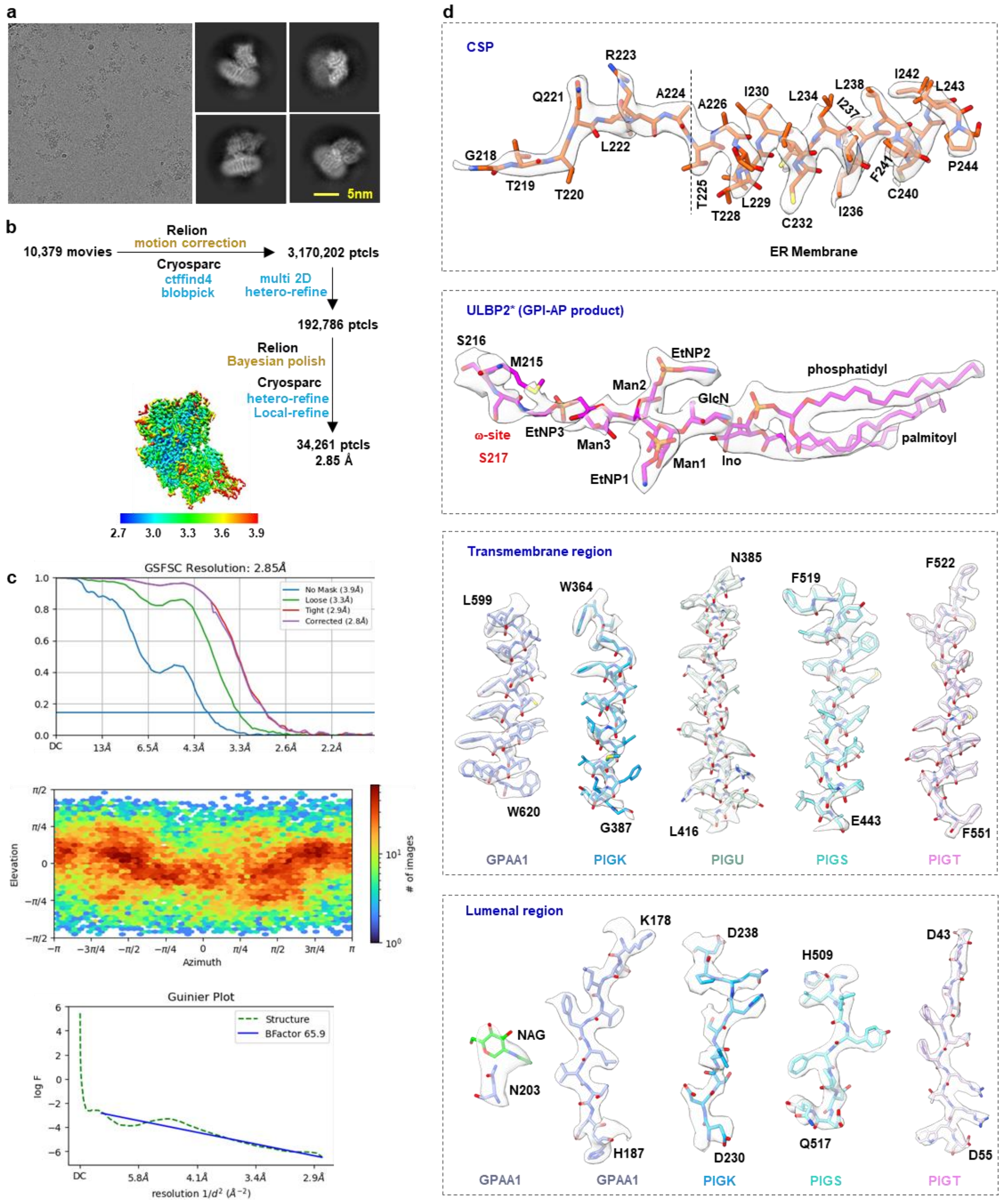

Supplementary Figure 5 | Cryo-EM data processing and density/model fitting exemplary views for GPI-T^{prod}. a Representative cryo-EM micrograph of GPI-T^{prod} and selected 2D class averages. **b** Workflow of the classification and refinement. **c** The nominal resolution of GPI-T was determined by the 'gold-standard' FSC curve using the FSC=0.143 criterion, and angular distribution heatmap at the bottom panel calculated in Cryosparc. Local resolution evaluation was shown on right. **d** Cryo-EM map density and model of representative protein parts, the protein parts that are discussed in the main text, and products.

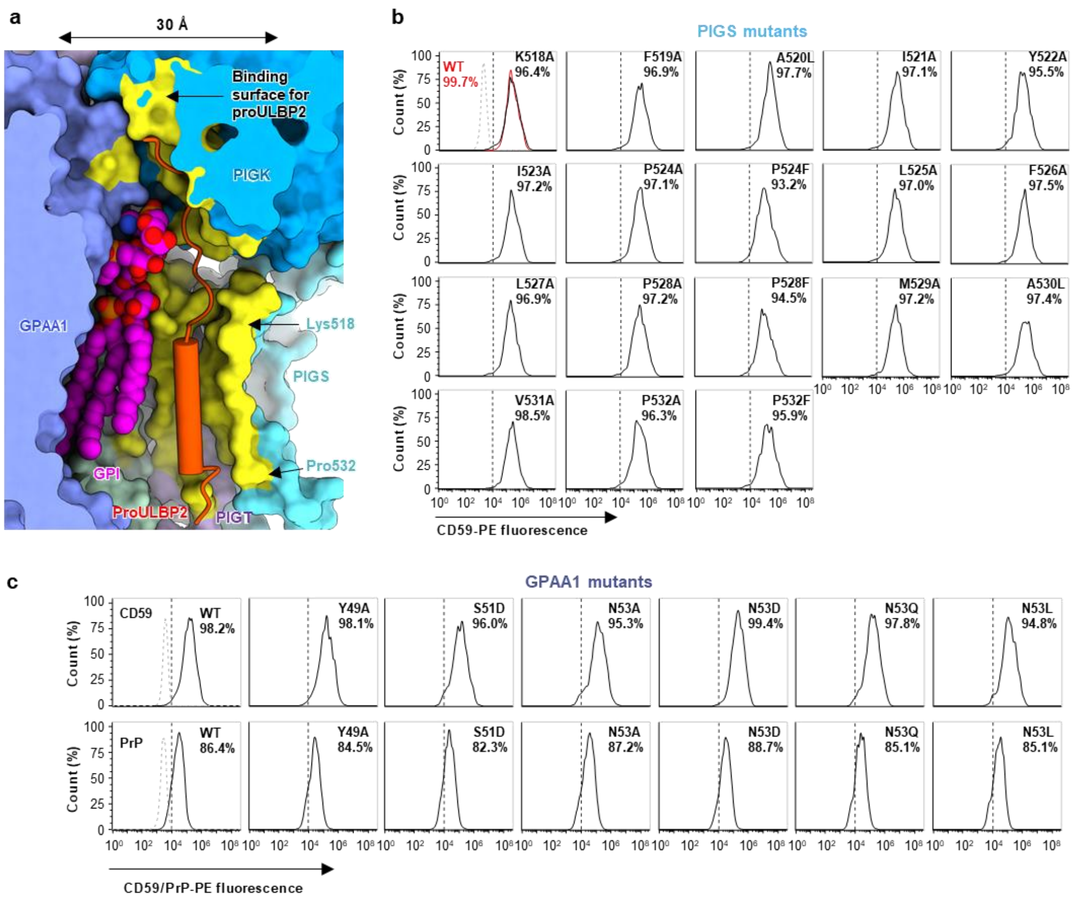

Supplementary Figure 6 | Characteristics of substrate binding. a An expanded cross-section view of the substrate binding site. GPI-T^{C206S} is shown in surface, proULBP2 in red cartoon, and GPI in magenta sphere. The binding footprint for proULBP2 is colored yellow. **b** The tolerance of the proULBP2-interacting PIGS TMH2 (**a**) to scanning mutagenesis. Plasmids carrying genes encoding the wild-type (WT) or the indicated mutants were transiently expressed in PIGS KO cells, and surface staining of CD59 was assessed by fluorescence-activated cell sorting. Source data are provided as a Source Data file. **c** Tolerance of three GPI-interacting GPAA1 residues to mutagenesis. Plasmids carrying genes encoding the wild-type (WT) or the indicated mutants were transiently expressed in GPAA1 KO cells, and surface staining of CD59 (top) and PrP (bottom) were assessed using fluorescence-activated cell sorting. The gating strategy for experiments in **b** and **c** is indicated in Supplementary Fig. 15b. Source data are provided as a Source Data file.

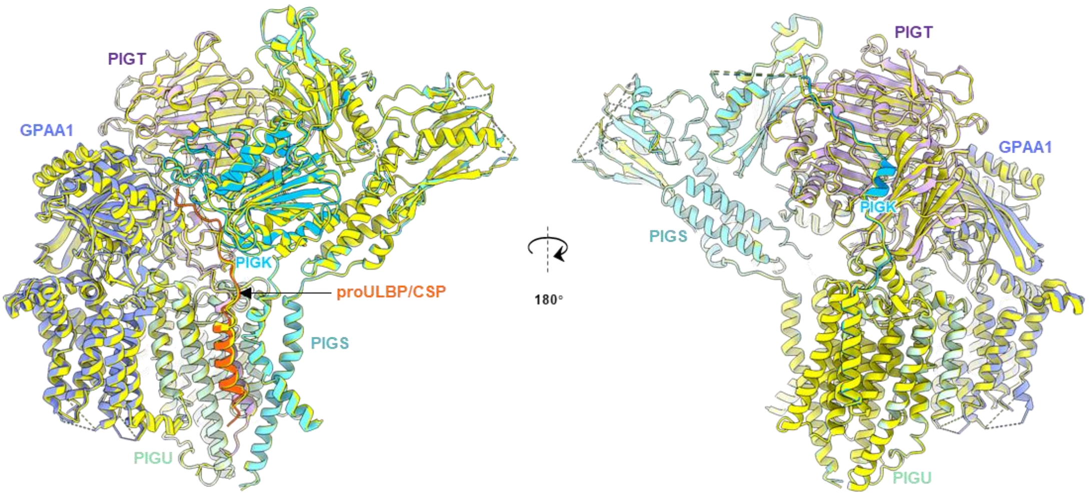

Supplementary Figure 7 | The overall similar structure of GPI-T^{sub} and GPI-T^{prod}. The products-bound GPI-T structure (yellow) is overall similar to the substrate-bound structure (other colors) with a Cα RMSD of 0.40 Å.

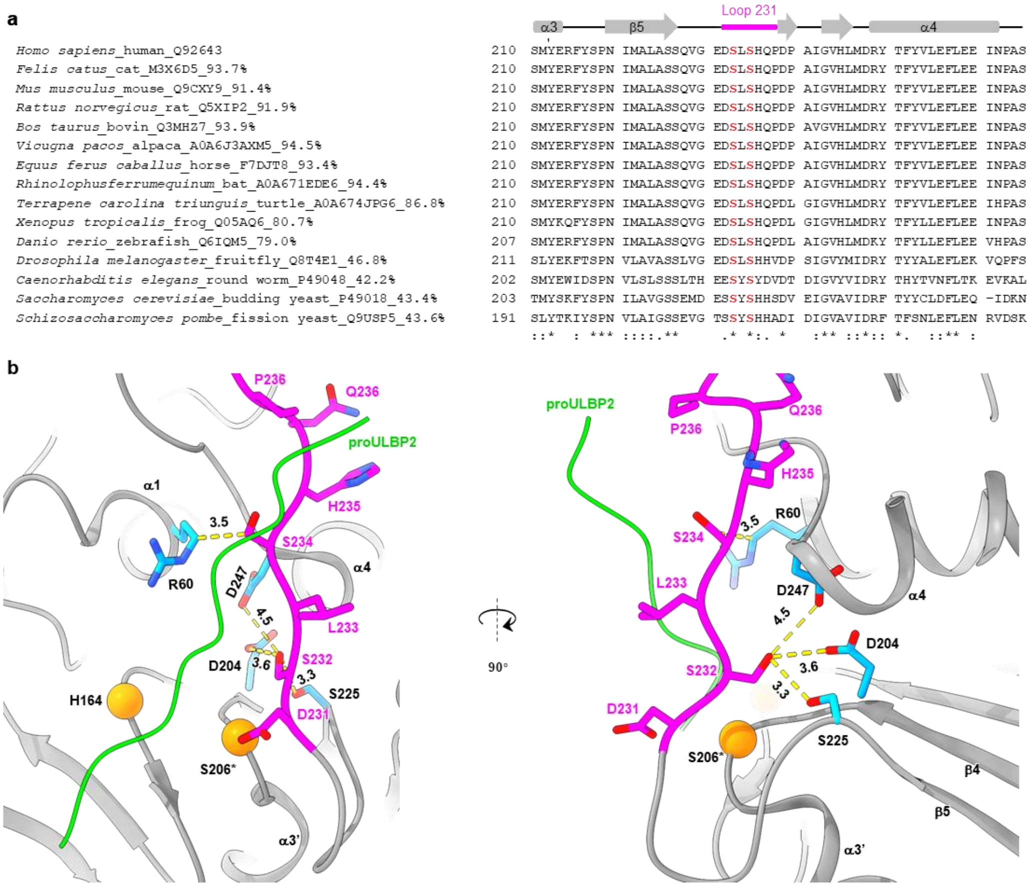

Supplementary Figure 8 | Sequence conservation of 231-Loop and the microenvironment of Ser232 and Ser234. a Sequence alignment of the 231-Loop region. Protein sequences were selected from evolutionarily representative species. Uniprot IDs and their identity to the human ortholog are shown alongside the binomial nomenclature and common names. Asterisk, colon, and dot indicate identical, conserved, and semi-conserved substitutions, respectively. Secondary structure elements are labeled as in Fig. 5b. **b** Structural rationale for the design of loos-of-function mutants on Ser232 and Ser234. PIGK (grey) is shown for the region near the 231-Loop (magenta). ProULBP2 (green) and the mutated catalytic dyad (His164 and Ser206*) are displayed for orientation purposes. The distances (Å) between the serine residues and surrounding residues are indicated by dashed lines. The relatively tight packing of these residues is consistent with the intolerance for replacement with large side chains (Fig. 5c**)**.

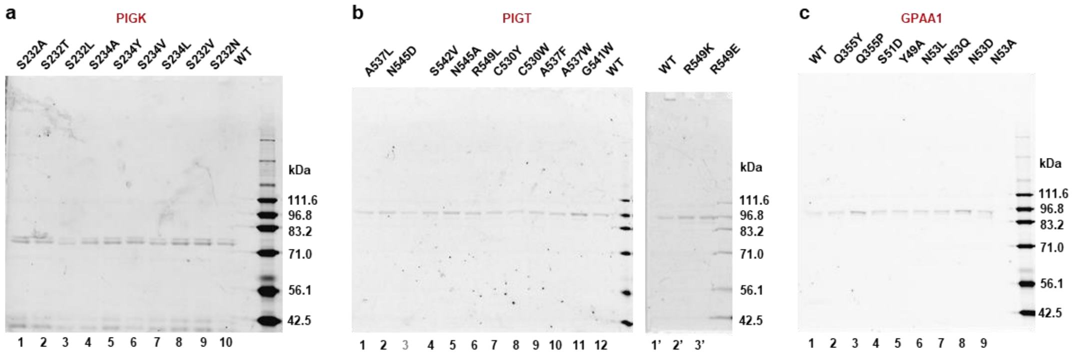

Supplementary Figure 9 | In-gel fluorescence of TGP-tagged subunits expressed in corresponding knockout (KO) cells. a-c Mutants of PIGK (**a**), PIGT (**b**), and GPAA1 (**c**) exhibit similar expression level to the wild-type (WT). Theoretical Molecular weights of the homemade GFP markers[4] are provided. Only the mutants reported in this paper are labeled. Low molecular-weight bands likely represent degradation products with an intact TGP tag. The in-gel fluorescence images were captured using the software Image Reader FLA-9000 Ver.1.0 on a Fujifilm FLA-9000 gel scanner. TGP, thermostable green fluorescence protein.

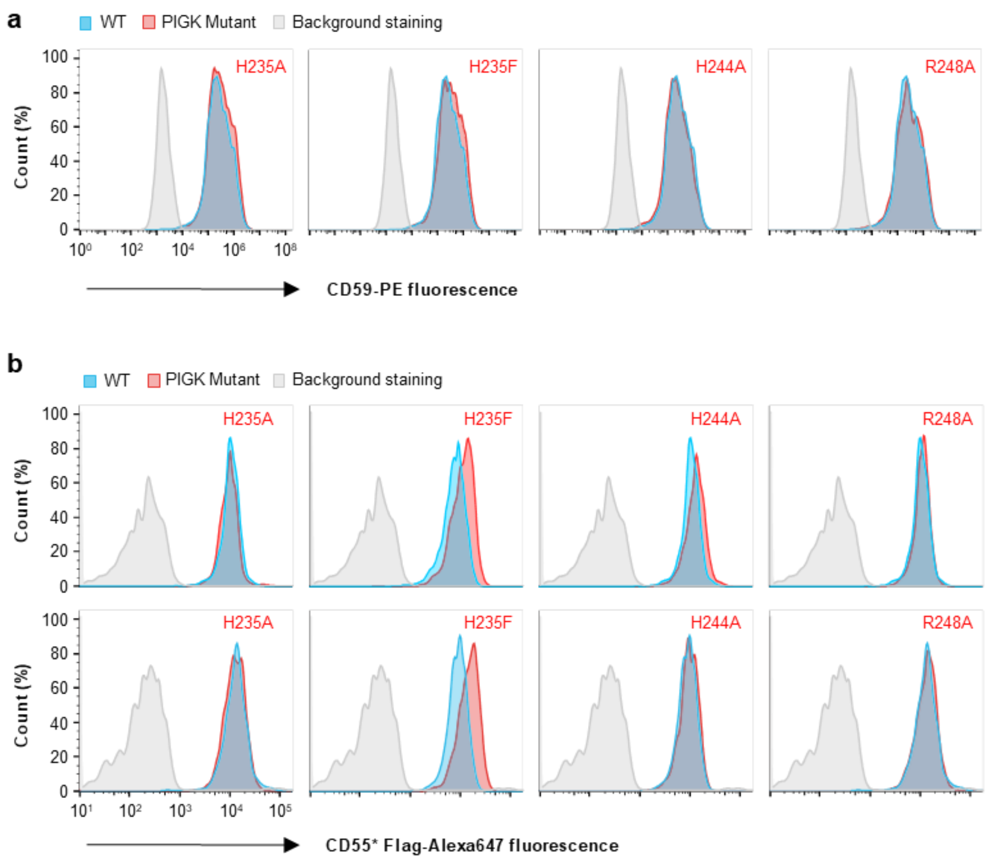

Supplementary Figure 10 | Apparent activity of mutant GPI-T containing substitutions of PIGK residues implicated in the auto-inhibition mechanism. a The surface expression of the endogenous GPI-AP marker (CD59) in PIGK-KO cells transfected with the wild-type PIGK (cyan), PIGK mutants (red), and an irrelevant membrane protein (grey) was assessed by fluorescence-activated cell sorting (FACS) using the gating strategy illustrated in Supplementary Fig. 15b. **b** The same as in **a** except that the marker was an overexpressed chimera GPI-AP (CD55*, Supplementary Fig. 2). See Supplementary Fig. 15c for gating strategy.

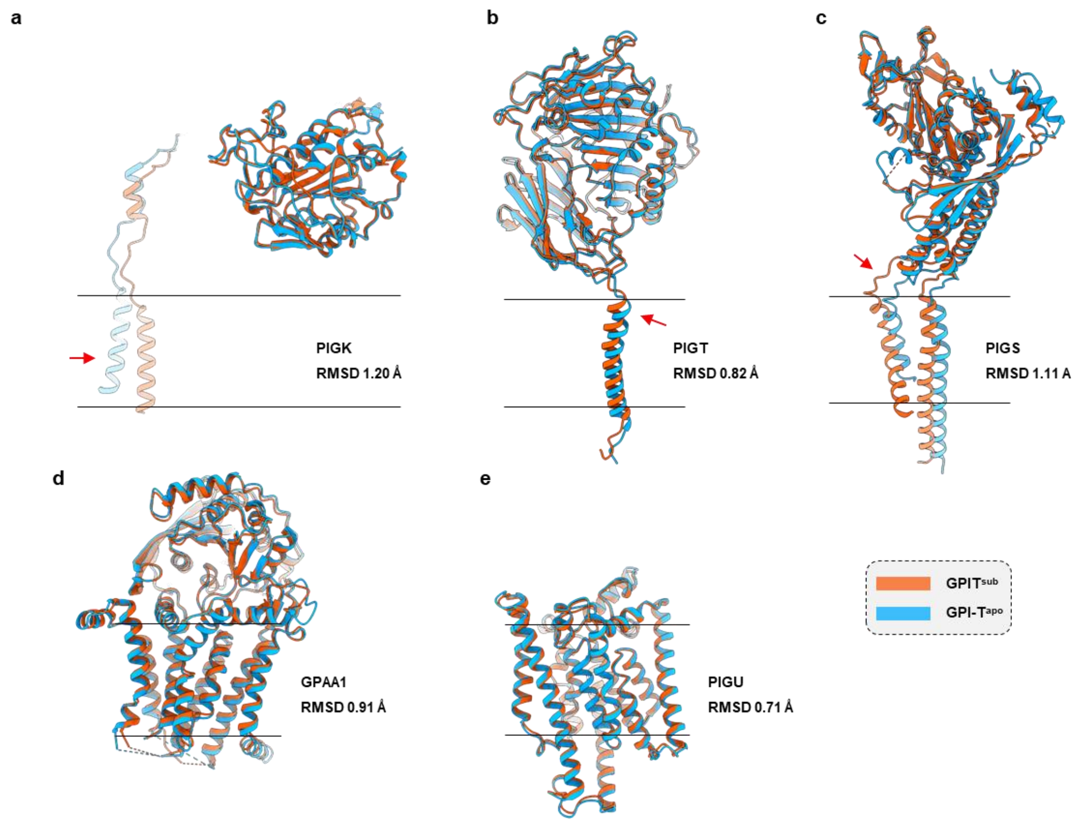

Supplementary Figure 11 | Conformational differences in individual subunits between GPI-T^{sub} and GPI-T^{apo}. a-e Structural alignment for PIGK (**a**), PIGT (**b**), PIGS (**c**), GPAA1 (**d**), and PIGU (**e**). ProULBP2- GPI-T^{sub} subunits (this work) are colored orange and proprotein-free GPI-T^{apo} subunits are colored blue (from PDB ID 7WLD[5] [http://doi.org/10.2210/pdb7WLD/pdb]). Cα RMSD values are indicated in each panel. Red arrows indicate relatively large conformational changes. Membrane boundaries are marked by the horizontal lines.

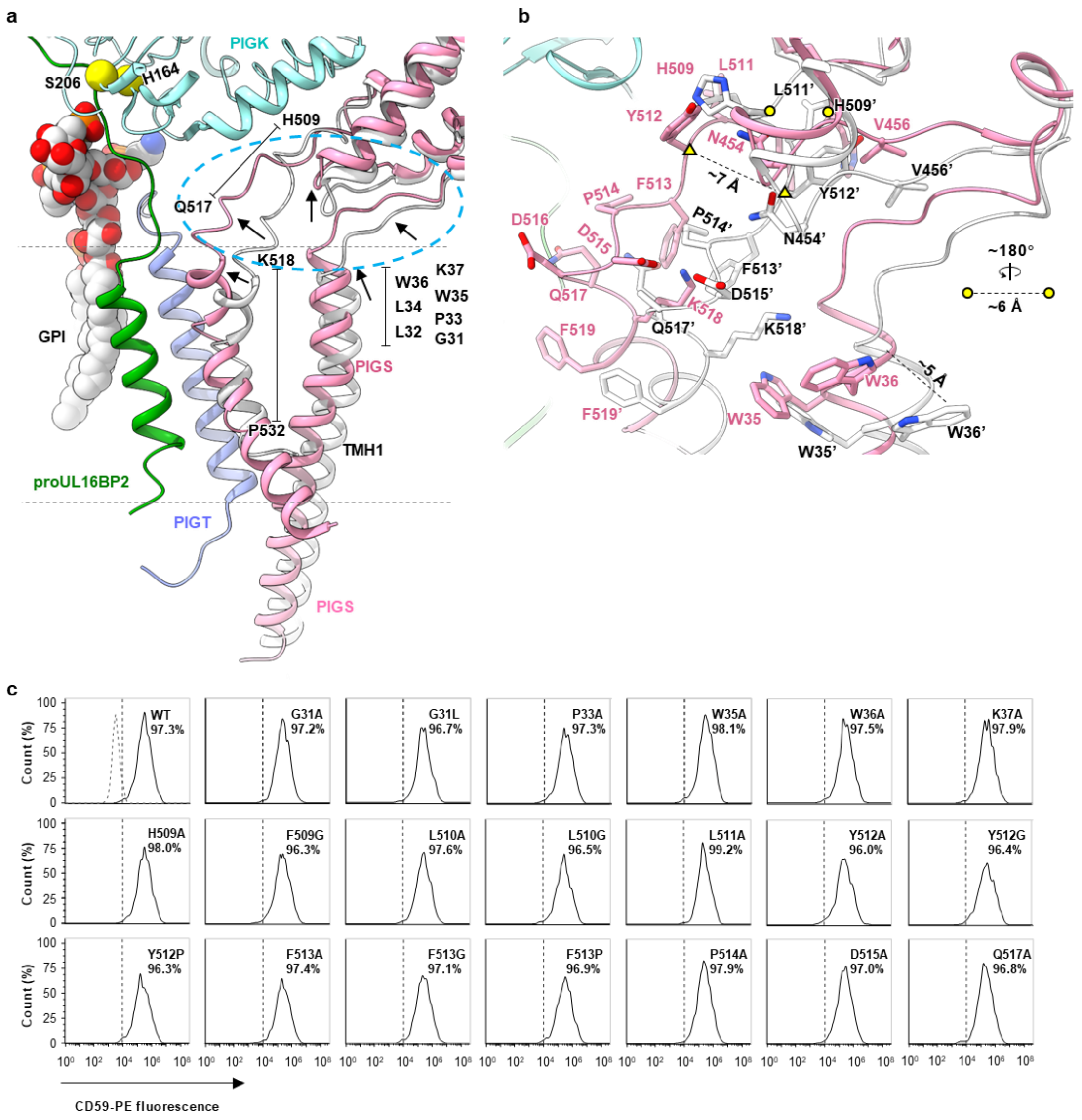

Supplementary Figure 12 | Conformational changes in PIGS and mutagenesis results of residues in this region. a Structural alignment of PIGS from GPI-T^{sub} (pink, this work) and GPI-T^{apo} (white, PDB ID 7WLD [http://doi.org/10.2210/pdb7WLD/pdb]) at and near the transmembrane region. Parts of PIGT (blue), PIGK (cyan), proULBP2 (green), the GPI molecule (white, sphere), and the Cα of the mutated catalytic dyad (yellow sphere) are displayed for orientation purposes. Arrows mark regions with relatively large conformational changes. Three line segments indicate residues that were systematically mutated. TMH, transmembrane helix. **b** An expanded view of the structural comparison of the juxtamembrane region as circled in **a**. Cα of residues with relatively large movements are highlighted with yellow cycles and triangles. Residue numbers from GPI-T^{apo} are

labeled with a prime, and those from GPI-T^{sub} are colored pink. **c** Tolerance of the PIGS residues to mutagenesis. Plasmids carrying genes encoding the wild-type (WT) or the indicated mutants were transiently expressed in PIGS KO cells and CD59 staining was performed using fluorescence-activated cell sorting with the gating strategy illustrated in Supplementary Fig. 15b. Results for residues ranging from Lys518 to Pro532 are in Supplementary Fig. 6b. Source data are provided as a Source Data file.

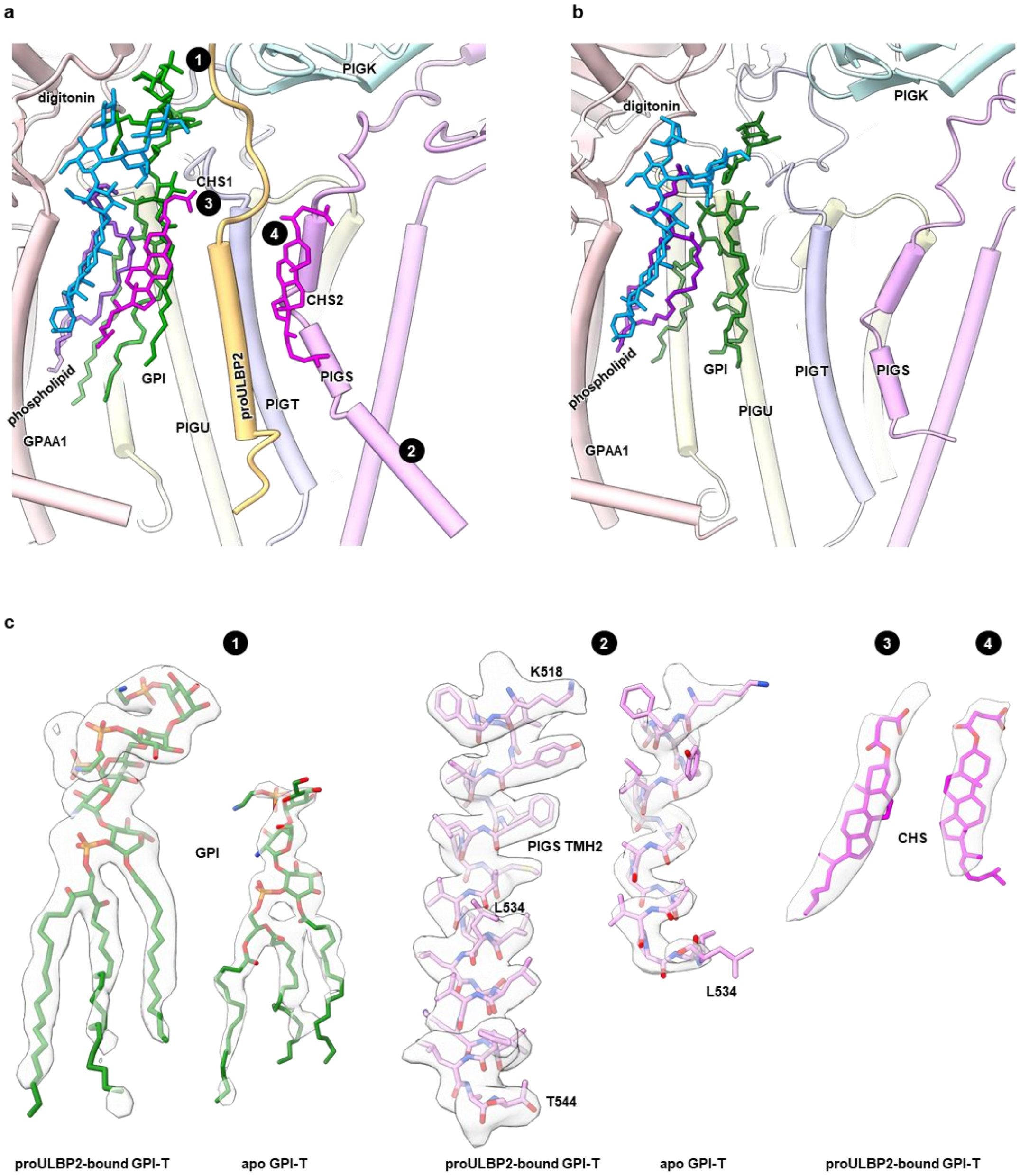

Supplementary Figure 13 | ProULBP2-binding induces orderliness of lipids and protein elements in the cavity. a The cavity of proULBP2-bound GPI-T^{sub}. CHS, cholesteryl hemisuccinate. **b** The cavity of proprotein-free GPI-T^{apo}. Major differences between the two structures are marked with numbers. **c** Cryo-EM density maps for the elements that show the described differences. Relevant components are appropriately labeled.

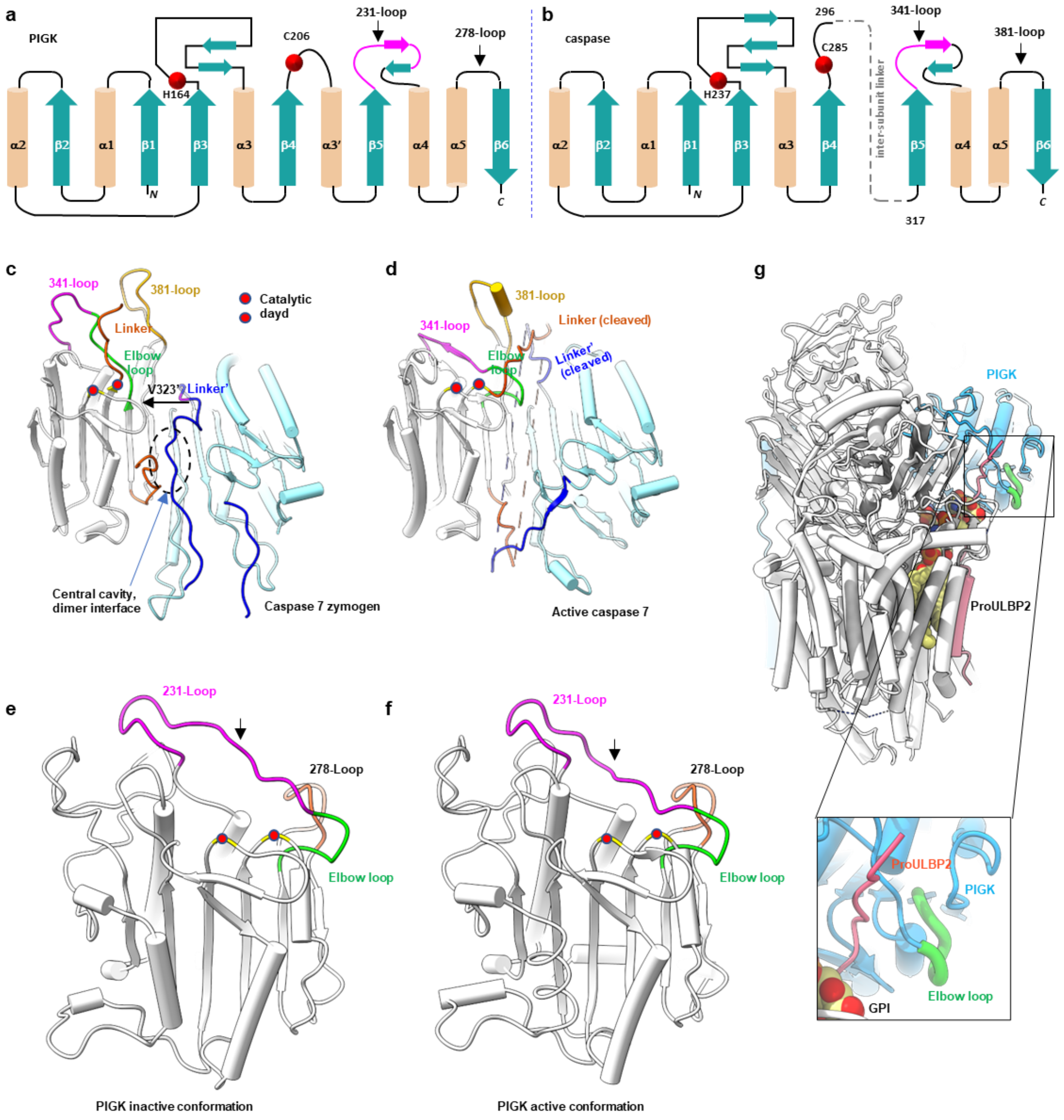

Supplementary Figure 14 | Similarity and difference of the activation mechanism between GPI-T and caspases. a, b PIGK (**a**) and caspases (**b**) show similar topological arrangements. Elements discussed in the texts are marked. **c, d** Conformational changes between the inactive state (**c**) and the active state (**d**) of caspase 7. In the inactive state, the inter-subunit linker located in the central cavity pushes the elbow loop, forcing the 341-Loop to assume a "lifted" conformation and block the active site. Proteolytic removal of the inter-subunit linker frees space for the elbow loop, allowing the re-shaping of the active site by the 341-Loop. **e, f** The conformational changes of PIGK during activation. The corresponding elbow loop and the 278-Loop (structural equivalent of the 381-Loop in caspases) remain

unchanged in the inactive (**e**) and active (**f**) states. Instead, the 231-Loop undergoes a "downward" movement. **g** Further structural differences between PIGK and caspases. The elbow loop is an important structural element in caspases to mediate the "push" by the inter-subunit linker in the inactive state. However, in PIGK, the elbow loop protrudes to bulk solvents and therefore is unlikely pushed by other GPI-T components.

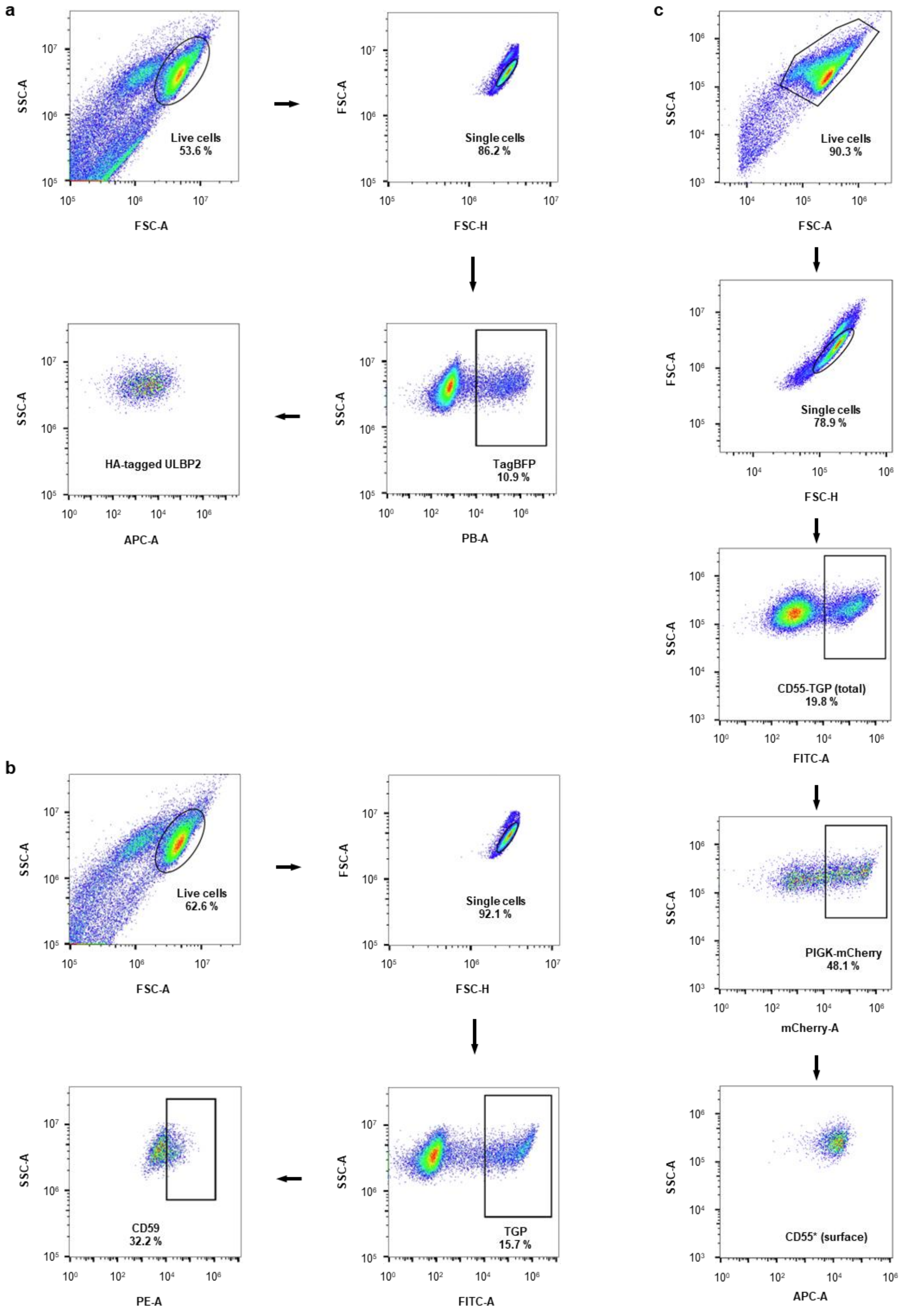

Supplementary Figure 15 | Gating strategies for fluorescence-activated cell sorting. a The cells were first gated to select living cells and single cells. The

expression of HA-tagged ULBP2 was gated by the fluorescence of the tagBFP (blue fluorescence protein) which was co-expressed with ULBP2 in an IRES-based construct (Supplementary Fig. 2a i). This population was further analyzed for the APC-positivity as an indication of the surface staining of ULBP2 via its HA-tag (Fig. 1b, Supplementary Fig. 3a). **b** TGP fused subunits (wild-type or mutants) were expressed in corresponding knockout cells. Apparent activity was assessed based on the surface staining of two endogenous GPI-AP reporters: CD59 and PrP. The cells were gated by TGP fluorescence to select the cells expressing TGP-fused subunits. The TGP-positive subpopulation was subsequently analyzed for the PE-positivity as an indication of the surface staining of the CD59/PrP/ULBP2* (Figs. 2c, 3c, 5c, Supplementary Fig. 3d, 6b, 6c, 10a, 12c). Only the representative data for CD59 are shown here. **c** PIGK-KO cells were co-transfected with plasmids encoding PIGK (wild-type or mutants) and the chimera CD55-TGP GPI-AP (CD55*) (Supplementary Fig. 2). Cells were first gated by the TGP fluorescence for the expression of the chimera protein. The TGP-positive population was further gated by mCherry fluorescence to select cells expressing PIGK. Finally, APC fluorescence (from anti-Flag antibodies) was used to gate cells having surface expression of CD55* (Fig. 5f, Supplementary Fig. 10b). The data distribution is shown as a heat map with low counts in blue and high counts in red. APC, allophycocyanin; FSC A/H, forward scatter area/height; FITC, fluorescein isothiocyanate (channel used in FACS analysis which detects TGP); TGP, thermostable green fluorescence protein; SSC A, side scatter area. PB, pacific blue; PE, phycoerythrin (channel used for CD59/PrP staining).

Supplementary Table 1 | Cryo-EM data collection and refinement statistics.

	**GPI-T^{sub}**	**GPI-T^{prod}**
**Data collection and processing**		
Magnification	130k	130k
Voltage (kV)	300	300
Electron exposure (e$^-$/Å^2)	50	50
Defocus range (μm)	-1~-2	-1~-2
Pixel size (Å)	0.932	0.932
Symmetry imposed	C1	C1
Initial particle images (no.)	2,981,565	3,170,202
Final particle images (no.)	176,889	34,261
Map resolution (Å)	3.22	2.85
FSC threshold	0.143	0.143
Map resolution range (Å)	3.22 – 4.20	2.7 – 3.9
**Refinement**		
Model resolution (Å)	3.58	3.10
FSC threshold	0.5	0.5
Map sharpening *B* factor (Å^2)	139.4	65.9
**Model composition**		
Non-hydrogen atoms	19,987	17,980
Protein residues	2,393	2,399
Ligands	35	27
B factor (Å^2)		
Protein	55.28	83.25
Ligand	68.64	93.39
R.m.s. deviations		
Bond lengths (Å)	0.004	0.004
Bond angles (°)	0.714	0.630
Validation		
MolProbity score	1.40	1.81
Clashscore	7.14	7.35
Ramachandran plot		
Favored (%)	97.97	97.81
Allowed (%)	2.03	2.19
Outliers (%)	0.00	0.00

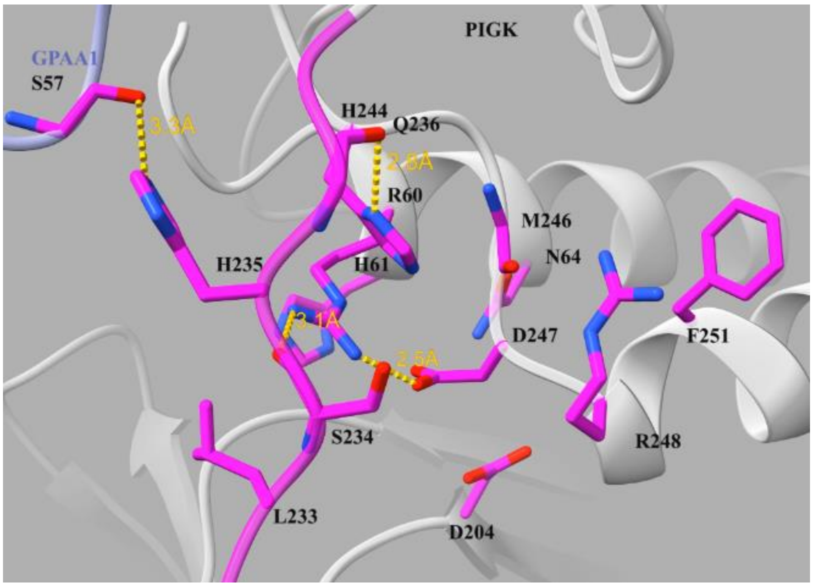

Supplementary Movie 1. Energetically unfavorable conformational changes during the activation of GPI-T. Hydrogen or ironic bonds were set to be broken at a distance of >3.8 Å. Interactions in the inactive state are indicated with orange dash lines and those in the active state with green dash lines. Sidechains are only shown if they are involved in the interactions. The movie was made using the "morph" function in ChimeraX.

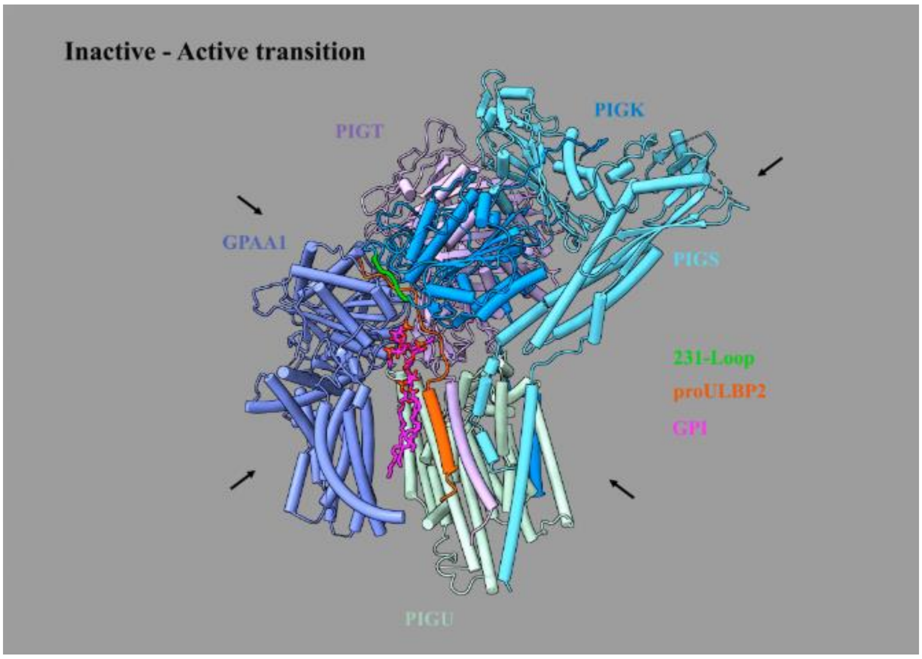

Supplementary Movie 2. Overall conformational changes upon proprotein binding. The movie was made using the "morph" function in ChimeraX. Prior to morphing, GPI-T^{apo} and GPI-T^{sub} were superimposed using the luminal domain of PIGK as the reference. In addition, the GPI molecule from GPI-T^{sub} was manually adjusted to best match the less-complete GPI molecule in GPI-T^{apo} as a rigid body.

Supplementary References

1. Wang, Y. et al. Genome-wide CRISPR screen reveals CLPTM1L as a lipid scramblase required for efficient glycosylphosphatidylinositol biosynthesis. *Proc Natl Acad Sci U S A* **119**, e2115083119 (2022).
2. Kinoshita, T. Biosynthesis and biology of mammalian GPI-anchored proteins. *Open Biol* **10**, 190290 (2020).
3. Kanzawa, N. et al. Peroxisome dependency of alkyl-containing GPI-anchor biosynthesis in the endoplasmic reticulum. *Proc Natl Acad Sci U S A* **106**, 17711-17716 (2009).
4. Cai, H., Yao, H., Li, T., Tang, Y. & Li, D. High-level heterologous expression of the human transmembrane sterol Δ8,Δ7-isomerase in Pichia pastoris. *Protein Expr Purif* **164**, 105463 (2019).
5. Xu, Y. et al. Molecular insights into biogenesis of glycosylphosphatidylinositol anchor proteins. *Nat Commun* **13**, 2617 (2022).
