# Supplementary material for: Structures of liganded glycosylphosphatidylinositol transamidase illuminate GPI-AP biogenesis
Source: Nat Commun. 2023 Sep 8;14:5520. doi: 10.1038/s41467-023-41281-y (PMC10491789; doi:10.1038/s41467-023-41281-y)
Supplement: Supplementary file 2 — Reporting Summary [file 41467_2023_41281_MOESM2_ESM.pdf]

nature portfolio

Corresponding author(s): Dianfan Li, Qianhui Qu

Last updated by author(s): Aug 11, 2023

Reporting Summary

Nature Portfolio wishes to improve the reproducibility of the work that we publish. This form provides structure for consistency and transparency in reporting. For further information on Nature Portfolio policies, see our Editorial Policies and the Editorial Policy Checklist.

Statistics

For all statistical analyses, confirm that the following items are present in the figure legend, table legend, main text, or Methods section.

n/a	Confirmed	
☐	☒	The exact sample size (*n*) for each experimental group/condition, given as a discrete number and unit of measurement
☐	☒	A statement on whether measurements were taken from distinct samples or whether the same sample was measured repeatedly
☒	☐	The statistical test(s) used AND whether they are one- or two-sided<br>*Only common tests should be described solely by name; describe more complex techniques in the Methods section.*
☒	☐	A description of all covariates tested
☒	☐	A description of any assumptions or corrections, such as tests of normality and adjustment for multiple comparisons
☐	☒	A full description of the statistical parameters including central tendency (e.g. means) or other basic estimates (e.g. regression coefficient) AND variation (e.g. standard deviation) or associated estimates of uncertainty (e.g. confidence intervals)
☒	☐	For null hypothesis testing, the test statistic (e.g. *F*, *t*, *r*) with confidence intervals, effect sizes, degrees of freedom and *P* value noted<br>*Give P values as exact values whenever suitable.*
☒	☐	For Bayesian analysis, information on the choice of priors and Markov chain Monte Carlo settings
☒	☐	For hierarchical and complex designs, identification of the appropriate level for tests and full reporting of outcomes
☒	☐	Estimates of effect sizes (e.g. Cohen's *d*, Pearson's *r*), indicating how they were calculated

Our web collection on statistics for biologists contains articles on many of the points above.

Software and code

Policy information about availability of computer code

Data collection	Preparative size exclusion chromatography, ChromLab 3.3.0.09; Cryo-EM data collection, EPU; In-gel fluorescence, TGreen Transilluminator OSE-470 (Fig. S3f), In-gel fluorescence, FLA-9000, Image Reader FLA-9000 Ver.1.0 (Fig. S9) ; Flow cytometry, Beckman CytoFlex LX, CytExpert 2.4.0.28, BD FACSAria Fusion, BD FACS Diva 8.0.3.
Data analysis	Cryo-EM data processing, Relion 3.1, CTFFIND4, and CryoSPARC 4.2.0; 3D-model building, Coot 0.9.6; Structure refinement, Phenix 1.19.2-4158; Structure visualization, PyMOL 2.3.3 and ChimeraX1.1; FACS data analysis, FlowJo v10.0.7.

For manuscripts utilizing custom algorithms or software that are central to the research but not yet described in published literature, software must be made available to editors and reviewers. We strongly encourage code deposition in a community repository (e.g. GitHub). See the Nature Portfolio guidelines for submitting code & software for further information.

Data

Policy information about availability of data

All manuscripts must include a data availability statement. This statement should provide the following information, where applicable:

- Accession codes, unique identifiers, or web links for publicly available datasets
- A description of any restrictions on data availability
- For clinical datasets or third party data, please ensure that the statement adheres to our policy

The coordinates for the model GPI-Tsub and GPI-Tprod generated in this study have been deposited in the PDB under accession code 8IMY [http://doi.org/10.2210/pdb8IMY/pdb] and 8IMX [http://doi.org/10.2210/pdb8IMX/pdb], respectively. The cryo-EM density maps for the GPI-Tsub and GPI-Tprod generated in this study

have been deposited in the Electron Microscopy Data Bank with accession code EMD- 35576 [https://www.ebi.ac.uk/pdbe/entry/emdb/EMD-35576] and 35575 [https://www.ebi.ac.uk/pdbe/entry/emdb/EMD-35575]. The coordinates for the previously published model GPI-Tapo are fetched from the PDB database under accession code 7WLD [http://doi.org/10.2210/pdb7WLD/pdb]. Uncropped images of Supplementary Fig. 3f, and tabular data for Figs. 2c, 3c, 5c, and Supplementary Figs. 6b, 6c and 12c are provided in the Source Data file.

Research involving human participants, their data, or biological material

Policy information about studies with human participants or human data. See also policy information about sex, gender (identity/presentation), and sexual orientation and race, ethnicity and racism.

Reporting on sex and gender	n/a
Reporting on race, ethnicity, or other socially relevant groupings	n/a
Population characteristics	n/a
Recruitment	n/a
Ethics oversight	n/a

Note that full information on the approval of the study protocol must also be provided in the manuscript.

Field-specific reporting

Please select the one below that is the best fit for your research. If you are not sure, read the appropriate sections before making your selection.

☒ Life sciences ☐ Behavioural & social sciences ☐ Ecological, evolutionary & environmental sciences

For a reference copy of the document with all sections, see nature.com/documents/nr-reporting-summary-flat.pdf

Life sciences study design

All studies must disclose on these points even when the disclosure is negative.

Sample size	The sample size (n=3) is stated in the figure legends. Sample size was chosen based on previous experience and similar reports in the literature (Nat Commun 2022 13:2617). No statistical methods were used to predetermine sample size.
Data exclusions	No data were excluded from the analysis.
Replication	All experimental results in this study are either from three independent experiments, or presented as a typical of at least three experiments. Similar results were obtained in repeated experiments using different cell batches and different plasmid preps and attempts to repeat the experiments were successful. Use of statistical methods have been described in relevant figure legends.
Randomization	The FACS assays sample a large number of cells that are from a single colony. Randomization was not relevant for this study. Biochemical and biophysical experiments, including protein purification, SDS-PAGE, and cryo-EM data collection, are not subjective and hence do not require randomization.
Blinding	Because the data collection and analysis procedures were not subjective, there was no need for blinding.

Reporting for specific materials, systems and methods

We require information from authors about some types of materials, experimental systems and methods used in many studies. Here, indicate whether each material, system or method listed is relevant to your study. If you are not sure if a list item applies to your research, read the appropriate section before selecting a response.

Materials & experimental systems

n/a	Involved in the study	
☐	☒	Antibodies
☐	☒	Eukaryotic cell lines
☒	☐	Palaeontology and archaeology
☒	☐	Animals and other organisms
☒	☐	Clinical data
☒	☐	Dual use research of concern
☒	☐	Plants

Methods

n/a	Involved in the study	
☒	☐	ChIP-seq
☐	☒	Flow cytometry
☒	☐	MRI-based neuroimaging

Antibodies

Antibodies used	Phycoerythrin (PE)-labeled CD59 antibody (12-0596-42, clone OV9A2, Thermo Fisher Scientific, 1 : 500 dilution); PE-labeled CD230 (PrP) Monoclonal Antibody (4D5) (12-9230-42, clone 4D5, Thermo Fisher Scientific, 1 : 50 dilution); Alexa Fluor® 647 Conjugated HA-Tag (6E2) Mouse mAb (Cat. 3444S, Cell Signaling Technology, 1:100 dilution); PE-labeled Myc-Tag (9B11) Mouse mAb (Cat. 3739S, Cell Signaling Technology, 1:300 dilution); Myc-tagged TGP-specific nanobody (Sb44) (Home-made, 0.02 mg/mL, Addgene #159421, Commun Biol. 2020; 3: 753); Alexa Fluor 647 conjugated Flag antibody (D6W5B) Rabbit mAb (Cat.15009S, Cell Signaling Technology, 1:100 dilution).
Validation	The validation of the Phycoerythrin (PE)-labeled CD59 antibody is conducted by the manufacturer. This OV9A2 monoclonal antibody reacts with human CD59, which is validated by staining the normal human peripheral blood cells with Mouse IgG1 K Isotype Control PE (Product # 12-4714-81) as the control and then analyzing with FACS. The information can be found with the link: https://www.thermofisher.cn/order/genome-database/dataSheetPdf?producttype=antibody&productsubtype=antibody_primary&productId=12-0596-42&version=216.<br><br>The validation of the PE-labeled CD230 (PrP) Monoclonal Antibody (4D5) is conducted by the manufacturer. This 4D5 antibody has been reported for use in flow cytometric analysis. This 4D5 antibody reacts with human CD59, which is validated by staining the normal human peripheral blood cells with Mouse IgG1 K Isotype Control PE (Product # 12-4714-81) as the control. The information can be found with the link: https://www.thermofisher.cn/order/genome-database/dataSheetPdf?producttype=antibody&productsubtype=antibody_primary&productId=12-9230-42&version=302<br><br>The validation of the Alexa Fluor® 647 Conjugated HA-Tag (6E2) Mouse mAb is conducted by the manufacturer. This 6E2 antibody is conjugated to Alexa Fluor® 647 fluorescent dye and tested in-house for direct flow cytometry and immunofluorescent analysis in cells transfected with HA-tagged protein. HA-Tag (6E2) Mouse mAb detects recombinant proteins containing the HA epitope tag. The antibody recognizes the HA-tag fused to either the amino or carboxy terminus of targeted proteins in transfected cells. The information can be found with the link: https://www.cellsignal.com/products/antibody-conjugates/ha-tag-6e2-mouse-mab-alexa-fluor-647-conjugate/3444<br><br>The validation of the PE-labeled Myc-Tag (9B11) Mouse mAb is conducted by the manufacturer. This 9B11 antibody is conjugated to phycoerythrin (PE) and tested in-house for direct flow cytometry in cells transfected with Myc-tagged protein. Myc-Tag (9B11) Mouse mAb (PE Conjugate) detects exogenously expressed proteins containing the Myc epitope tag. This antibody recognizes the Myc tag fused to either the amino or carboxy terminus of targeted proteins in transfected cells. Myc-Tag (9B11) Mouse mAb (PE Conjugate) detects exogenously expressed Myc-tagged proteins in cells expressed under a CMV promoter. Expression under other promoters has not been evaluated. The antibody may cross-react with c-myc protein. The information can be found with the link: https://www.cellsignal.com/products/antibody-conjugates/myc-tag-9b11-mouse-mab-pe-conjugate/3739<br><br>The Myc-tagged TGP-specific nanobody (Sb44) has a binding affinity (Kd) of 4.4 nM (Addgene #159421, Commun Biol. 2020; 3: 753)<br><br>The validation of the Alexa Fluor 647 conjugated Flag antibody (D6W5B) is conducted by the manufacturer. This D6W5B antibody is conjugated to Alexa Fluor® 647 fluorescent dye and tested in-house for direct flow cytometry analysis in monkey cells. This antibody is expected to exhibit the same species cross-reactivity as the unconjugated DYKDDDDK Tag (D6W5B) Rabbit mAb (Binds to same epitope as Sigma's Anti-FLAG® M2 Antibody) #14793. DYKDDDDK Tag (D6W5B) Rabbit mAb (Binds to same epitope as Sigma's Anti-FLAG® M2 Antibody) (Alexa Fluor® 647 Conjugate) detects exogenously expressed DYKDDDDK proteins in cells. The antibody recognizes the DYKDDDDK peptide, which is the same epitope recognized by Sigma's Anti-FLAG® antibodies, fused to either the amino-terminus or carboxy-terminus of the target protein. The information can be found with the link: https://www.cellsignal.com/products/antibody-conjugates/dykddddk-tag-d6w5b-rabbit-mab-binds-to-same-epitope-as-sigma-s-anti-flag-m2-antibody-alexa-fluor-647-conjugate/15009.

Eukaryotic cell lines

Policy information about cell lines and Sex and Gender in Research

Cell line source(s)	HEK293 cells, ATCC (Cat. CRL-3216); GPI-T single subunit knock-out cell lines (GPAA1, PIGK, PIGS, PIGT), generated in our previous study (Nat Commun 2022 13: 2617); PGAP1 knock-out cell line, generated in this study.
Authentication	The knock-out cell lines were verified using PCR, sequencing, and FACS analysis. HEK-293 cells were not authenticated. Cells were maintained at lowest passage numbers possible to maintain identity.
Mycoplasma contamination	The cell lines were not tested for mycoplasma contamination.
Commonly misidentified lines (See ICLAC register)	No commonly misidentified cell lines were used in this study.

Flow Cytometry

Plots

Confirm that:

- [x] The axis labels state the marker and fluorochrome used (e.g. CD4-FITC).
- [x] The axis scales are clearly visible. Include numbers along axes only for bottom left plot of group (a 'group' is an analysis of identical markers).
- [x] All plots are contour plots with outliers or pseudocolor plots.
- [x] A numerical value for number of cells or percentage (with statistics) is provided.

Methodology

Sample preparation	Transfected wild-type or GPI-T single sununit KO HEK293 cells were washed with PBS, treated with trypsin, and washed and resuspended in 0.5 mL PBS. Phycoerythrin (PE)-labeled CD59 antibody (12-0596-42, Thermo Fisher Scientific) or prion antibody (Cat. 12-9230-42, Thermo Fisher Scientific) were used as a 500-fold (CD59) or 50-fold (prion) dilution for incubation with the cells for 15 min at dark. Cells were washed with PBS and resuspended in ~0.3 mL of PBS for flow cytometry (Beckman CytoFlex LX) monitored at two wavelength pairs (488/525 for GFP, 561/585 for PE).<br>For surface staining of HA-tagged ULBP2, HEK293 cells or PIGK-KO HEK293 cells transfected with pULBP2(FACS) were treated the same way as mentioned above. To the resuspended cells, Alexa Fluor 647 conjugated HA-Tag antibody (Cat. 3444S, Cell Signaling Technology, 1:100 dilution) was incubated with the cells for 15 min in dark. Cells were rinsed with PBS and resuspended in ~0.3 mL of PBS for flow cytometry (Beckman CytoFlex LX). Cells (typically 60,000) were gated using the TagBFP channel (405/450 nm) and the Alexa Fluor 647 channel (638/660 nm) as reporters for successful transfection and surface expression, respectively.<br>For surface staining of ULBP2* (TGP-containing fluorescent GPI-AP), cells were incubated with or without 10 µg of PI-PLC for 1.5 h at 37 ºC. Cells were washed with PBS, and incubated with 10 µg of a TGP-specific nanobody (Sb44). After 15 min, unbound Sb44 were removed and a PE-conjugated second antibody (Cat. 3739S, Cell Signaling Technology, 1:300 dilution) recognizing the Myc-tag on Sb44 was then added for staining at RT for 15 min. After a rinse step, cells were subjected to FACS analysis as outlined above.<br>For surface staining of CD55* (TGP-CD55 chimera protein), PIGK-KO cells were co-transfected with pCD55chimera and the plasmid encoding a mCherry-tagged PIGK (wild-type, mutants, or an irrelevant membrane protein protein) using Lipofectamine 3000. The plasmid ratio for PIGK and CD55* was 1:1 (wt:wt). The rest of the procedures were the same as above. For surface staining, cells were resuspended in 0.1 mL Alexa Fluor 647 conjugated Flag antibody diluted in PBS (Cat.15009S, Cell Signaling Technology, 1:100) and incubated at RT for 30 min protected from light. Cells were washed once with PBS and resuspended in 0.5 mL of PBS for flow cytometry (Beckman, CytoFlex LX) monitored at three wavelength pairs (488/525 nm for TGP, 561/610 nm for mCherry, 638/660 nm for Alexa 647). Cells (typically 40,000) were gated using the GFP channel (from expression of CD55*), the mCherry channel (for expression of PIGK mutants or WT) and analyzed for signal for APC channel (for surface staining of Flag-tagged CD55*) using the software FlowJo (BD Life Sciences).
Instrument	Beckman CytoFlex LX
Software	CytExpert 2.4.0.28 was used to collect the flow cytometry data. FlowJo v10.0.7 was used to analyze the flow cytometry data.
Cell population abundance	The commercial HEK cell lines has a cell population of 100%. The knockout cell lines we generated in this study was developed from single colonies and also have a cell population of 100%. Typically 40,000 cells were analyzed for each sample.
Gating strategy	For the cell surface staining of CD59, prion and ULBP2* (TGP-containing fluorescent GPI-AP), cells were gated using the GFP channel (from expression of TGP-tagged GPI-T subunit(s) or ULBP2*) and analyzed for positive signal for the PE channel (for surface staining of CD59, prion or ULBP2*).<br>For surface staining of HA-tagged ULBP2, cells were gated using the TagBFP channel (405/450 nm) and the Alexa Fluor 647 channel (638/660 nm) as reporters for successful transfection and surface expression, respectively.<br>For surface staining of Flag-tagged CD55*, cells were gated using the GFP channel (from expression of CD55*), the mCherry channel (for expression of PIGK mutants or WT) and analyzed for signal for APC channel (for surface staining of Flag-tagged CD55*).

- [x] Tick this box to confirm that a figure exemplifying the gating strategy is provided in the Supplementary Information.
