# Supplementary material for: Structures of liganded glycosylphosphatidylinositol transamidase illuminate GPI-AP biogenesis
Source: Nat Commun. 2023 Sep 8;14:5520. doi: 10.1038/s41467-023-41281-y (PMC10491789; doi:10.1038/s41467-023-41281-y)
Supplement: Supplementary file 4 — Description of additional supplementary files [file 41467_2023_41281_MOESM4_ESM.pdf]

Description of Additional supplementary files

File Name: **Supplementary Movie 1**

Description: **Energetically unfavorable conformational changes during the activation of GPI-T.** Hydrogen or ironic bonds were set to be broken at a distance of >3.8 Å. Interactions in the inactive state are indicated with orange dash lines and those in the active state with green dash lines. Sidechains are only shown if they are involved in the interactions. The movie was made using the "morph" function in ChimeraX.

File Name: **Supplementary Movie 2**

Description: **Overall conformational changes upon proprotein binding.** The movie was made using the "morph" function in ChimeraX. Prior to morphing, GPI-T^{apo} and GPI-T^{sub} were superimposed using the luminal domain of PIGK as the reference. In addition, the GPI molecule from GPI-T^{sub} was manually adjusted to best match the less-complete GPI molecule in GPI-T^{apo} as a rigid body.
